# Does Exposure to Noise During Military Service Affect the Progression of Hearing Loss with Increasing Age?

**DOI:** 10.1177/23312165221076940

**Published:** 2022-02-07

**Authors:** Brian C. J. Moore, David A. Lowe

**Affiliations:** 1 Cambridge Hearing Group, Department of Psychology, University of Cambridge, Cambridge, UK; 2 ENT Department, 156705 James Cook University Hospital, Middlesbrough, Cleveland, UK

**Keywords:** noise exposure, military service, progression of hearing loss, noise-induced hearing loss

## Abstract

It is traditionally believed that the effects of exposure to noise cease once the exposure itself has ceased. If this is the case, exposure to noise relatively early in life, for example during military service, should not affect the subsequent progression of hearing loss. However, recent data from studies using animals suggest that noise exposure can accelerate the subsequent progression of hearing loss. This paper presents new longitudinal data obtained from 29 former male military personnel. Audiograms obtained at the end of military service were compared with those obtained at least five years later. Rates of change of hearing threshold level (HTL) in dB/year were compared with those expected from ISO7029 (2017) for men at the 50^th^ percentile. The results are consistent with the hypothesis that noise exposure during military service accelerates the progression of hearing loss for frequencies where the hearing loss is absent or mild at the end of military service, by about 1.7 dB/year on average for frequencies from 3 to 8 kHz, but has no effect on or slows the progression of hearing loss for frequencies where the hearing loss exceeds about 50 dB. Acceleration appears to occur over a wide frequency range, including 1 kHz. There remains a need for further longitudinal studies using larger sample sizes. Longitudinal studies are also needed to establish whether exposure to other types of sounds, for example at rock concerts or from work in heavy industries, affects the subsequent progression of hearing loss.

## Introduction

It is traditionally believed that the effects of exposure to noise cease once the exposure itself has ceased (Humes et al., 2006; Mirza et al., 2018). If this is the case, exposure to noise should not affect the progression of hearing loss with increasing age after the exposure ceases. Data from longitudinal studies of humans mostly support this common belief (Hederstierna & Rosenhall, 2016; Lee et al., 2005). However, as reviewed by Moore (2021), those studies were largely based on older people (aged 70 years or more), and even the non-noise exposed participants had substantial hearing loss at high frequencies. Furthermore, those studies included only a small proportion of military veterans; most of the noise-exposed individuals had worked in noisy factories. This paper addresses the issue of whether noise exposure during military service affects the progression of hearing loss following the end of military service.

The noise occuring in many noisy work places is relatively steady, and it is typically broadband with levels of 90–110 dB SPL. Prolonged exposure to such noise typically produces a “notch” or “bulge” in the audiogram for a frequency close to 4 kHz (Passchier-Vermeer, 1974; Smoorenburg, 1992). In contrast, military service often involves exposure to impulsive sounds from rifle shots, mortars, anti-tank weapons, and explosions, as well as exposure to more steady noises from vehicles and aircraft. The peak levels of the impulsive sounds encountered during military service can reach 155 dB SPL (Jokel et al., 2019). Furthermore, many military personnel report that they do not use hearing protection (or use it only loosely fitted) during active service (Lowe & Moore, 2021). For a given mean exposure level, impulsive sounds are more damaging to the ear than steady sounds (Henderson & Hamernik, 1986; Zhang et al., 2021). Thus, it seems reasonable to assume that the effects of noise exposure during military service may be different from the effects of exposure to steady factory noise. Consistent with this, noise exposure during military service often leads to greater hearing loss at 6 and 8 kHz than at 4 kHz (Lowe & Moore, 2021; Moore, 2021). Also, exposure to steady noise typically leads to hearing loss that is similar for the two ears (Passchier-Vermeer, 1974; Smoorenburg, 1992), while exposure to noise during military service often leads to greater hearing loss in one ear than the other, because of the asymmetric nature of the exposure (Keim, 1969; Lowe & Moore, 2021; Moore, 2020)

The possibility that noise exposure can accelerate the progression of hearing loss following the exposure is supported by studies using mice. Kujawa and Liberman (2006) compared the progression of hearing loss with increasing age for non-exposed mice and mice exposed to an octave-wide band of noise (8–16 kHz) with a level of 100 dB SPL for two hours. The age of the mice at the time of exposure varied from 4 to 124 weeks. Control and noise-exposed mice were housed together for post-exposure times from 2 to 96 weeks. When tested 2 weeks after exposure (using auditory brainstem responses, ABRs, to estimate detection thresholds), shifts in threshold up to 40–50 dB were found for animals that were exposed when young (4–8 weeks of age), but animals exposed at the age of 16 weeks or later showed almost no threshold shift. When tested a long time after the exposure, exposed animals, regardless of the age of exposure, showed greater hearing loss than age-matched non-exposed controls. The authors concluded that “Data suggest that pathologic but sublethal changes initiated by early noise exposure render the inner ears significantly more vulnerable to aging.”

In a similar study using mice (Fernandez et al., 2015), the effects of two levels of noise exposure were compared. The higher exposure (an octave-wide noise band for two hours at 100 dB SPL) produced permanent damage to the synapses between inner hair cells (IHCs) and primary auditory neurons (called synaptopathy, Kujawa and Liberman, 2009) without hair cell loss. The lower exposure (an octave-wide noise band for two hours at 91 dB SPL) produced neither synaptic damage nor hair cell loss. A control group with no exposure was also used. Cochlear function was assessed from 1 h to about 20 months after exposure via distortion product otoacoustic emissions (DPOAEs, which provide a measure of outer hair cell, OHC, function, Kemp, 1978) and ABRs. The 100 dB SPL noise led to threshold shifts of 35–50 dB 24 h after exposure. After two weeks, thresholds recovered, but synaptic counts and ABR amplitudes at high frequencies were reduced by up to 45%. About 20 months after exposure, thresholds were up to 18 dB greater for the group with synaptopathy than for the other two groups. OHC losses worsened over the same time frame. The group receiving the lower exposure did not show acceleration of synaptic loss or cochlear dysfunction with increasing age up to about 1 year after exposure. The authors concluded that “Therefore, interactions between noise and aging may require an acute synaptopathy, but a single synaptopathic exposure can accelerate cochlear aging”.

Moore (2021) argued that mild to moderate hearing loss is usually primarily a consequence of loss of function of the OHCs. A certain amount of damage to the OHCs can occur with little or no change in the detection threshold (Dallos & Harris, 1978; Evans & Harrison, 1976; Harrison & Evans, 1979). This is consistent with the concept of a “cochlear reserve”; the cochlea can sustain some damage without loss of function as revealed by the audiogram, but once the reserve is sufficiently depleted effects in the audiogram become apparent. If hearing loss approaching 55 dB is present at some frequencies at the end of a noise exposure, this could be due primarily to near-complete loss of function of OHCs. In this case, acceleration of the subsequent progression of hearing loss due to further OHC damage is not expected. In contrast, if the hearing loss at the end of noise exposure is slight or mild at some frequencies, then there is scope for acceleration of the subsequent progression of hearing loss at those frequencies due to further damage to OHCs. This led Moore (2021) to propose the following hypothesis: For frequencies where the noise-induced hearing loss (NIHL) at the end of military service is mild, exposure to noise during military service accelerates the subsequent progression of hearing loss. In contrast, for frequencies where the NIHL is moderate or severe at the end of military service, the prior noise exposure has little or no effect on or even slows the subsequent progression of hearing loss.

There are three published studies that are directly relevant to this issue. Macrae (1971, 1991) compared the hearing threshold levels (HTLs) of military veterans obtained close to the end of military service and after an interval of several years, for frequencies of 1 and 4 kHz. Moore (2021) conducted a re-analysis of those data. The rates of change of HTL following the end of military service were compared with those expected from ISO 7029 (2017), which is a current standard based on a large population who were carefully screened to exclude noise-exposed individuals. The rates of change of HTL reported by Macrae tended to increase with increasing age (and increasing hearing loss), but the progression was somewhat irregular. To smooth the data, a linear regression line was fitted to the rate of change as a function of age, and the fitted line was used to predict the rate of change for each age group. At 1 kHz, a frequency for which hearing loss at the end of military service was small or absent, the observed rate of change of HTL was greater than predicted from ISO 7029 (2017), regardless of age group. At 4 kHz, a frequency for which there was some hearing loss at the end of military service, the observed rate of change of HTL was greater than predicted from ISO 7029 (2017) for the younger age groups (who had on average small hearing losses) but was smaller than predicted from ISO 7029 (2017) for the older age groups (who had on average larger hearing losses). Moore (2021) concluded that the results were consistent with his hypothesis.

 The other two studies that are relevant to the hypothesis (Kim et al., 2017; Xiong et al., 2014) were both cross-sectional rather than longitudinal in design, which limits the conclusions that can be drawn. Also, both studies had some design limitations, as discussed by Moore (2021). Nevertheless, the results of both studies support the hypothesis that for frequencies where the NIHL at the end of military service is mild, exposure to noise during military service accelerates the subsequent progression of hearing loss.

The present paper presents new longitudinal data from former military personnel. Audiograms obtained at the end of military service were compared with those obtained at least five years later. Rates of change of HTL in dB/year were compared with those expected from ISO 7029 (2017) for men at the 50^th^ percentile.

## Longitudinal Study of Changes in HTL Following the End of Military Service

### Study Sample

**Figure 1.**
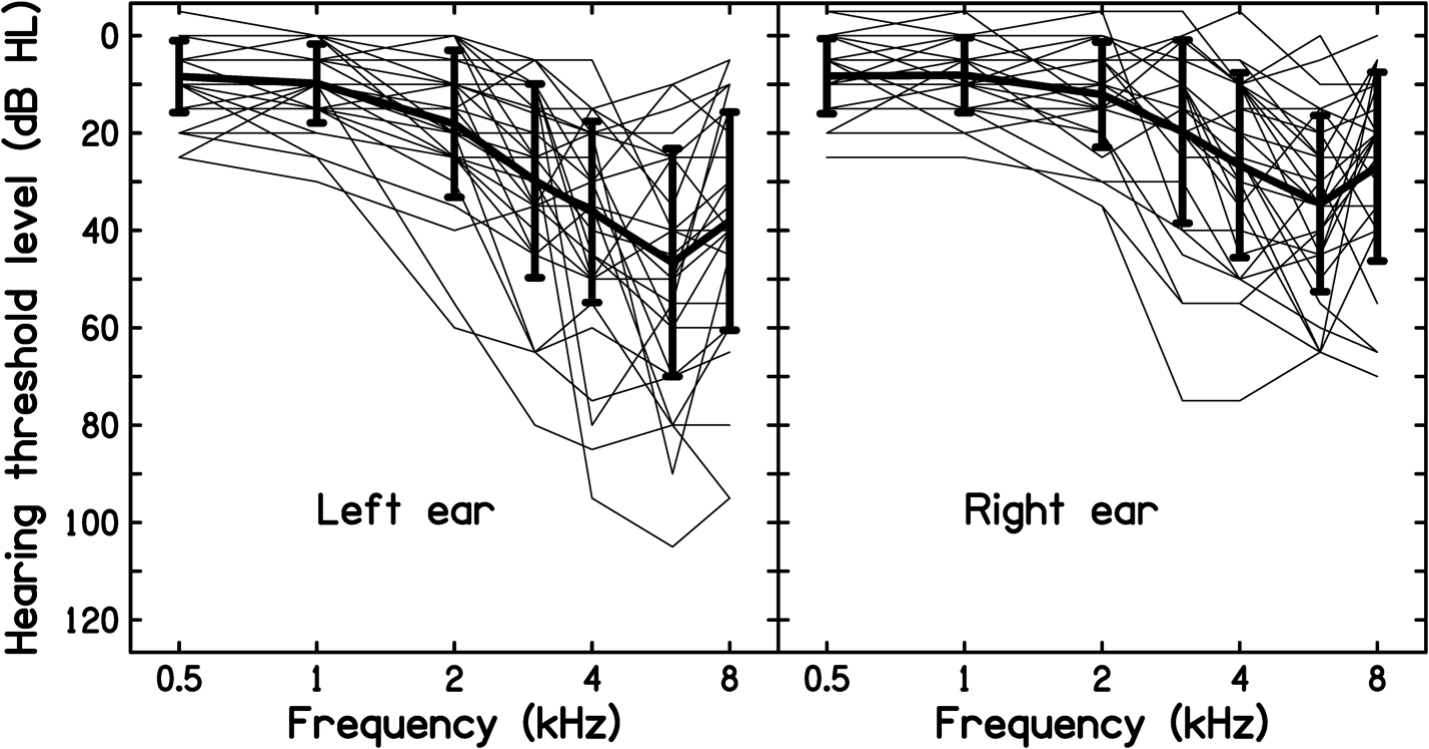
The thin lines show individual audiograms obtained close to the end of military service for the left and right ears. The thick lines show the means and error bars show ± 1 standard deviation.

Data were available for 29 UK male military veterans, most of whom had served in the army. Their age at entry to military service ranged from 16 to 24 years. Their age at the end of military service ranged from 23 to 44 years; 5 were aged 23–25, 6 were aged 26–30, 5 were aged 31–35, 4 were aged 36–40, and 9 were aged 41–44 years. All had HTLs better than or equal to 20 dB HL from 0.5 to 6 kHz at the start of military service (HTLs at 8 kHz were not measured for all cases). All were claiming compensation for NIHL. The claims were initiated from 5 to 20 years after the end of military service, with a mean of 10 years, a median of 10 years and a standard deviation of 6 years. They were selected from larger databases on the basis of reliable audiograms, obtained according to the standards of the British Society of Audiology (2018), being available near the end of military service and five or more years later. The time interval between the end-of-service audiogram and the later audiogram ranged from 5 to 27 years; the interval was 5–10 years for 8 men, 11–15 years for 9 men, 16–20 years for 6 men, 21–25 years for 4 men, and 26–27 years for 2 men. The men had a wide range of hearing losses at the end of military service for frequencies from 3 to 8 kHz. Individual and mean end-of-service audiograms are shown in Figure 1. These show similar features to those published previously for cases of NIHL incurred during military service, specifically a tendency for the greatest hearing loss to occur at 6 kHz and greater hearing loss for the left than for the right ears, on average (Lowe & Moore, 2021; Moore, 2020). None of the men showed any evidence of significant conductive hearing loss (air-bone gaps were 10 dB or less). None of the men had a history of exposure to ototoxic substances or medications, none had current or previous ear diseases, and none had a family history of ear disorders. All of the men reported exposure to intense impulsive sounds during military service, sometimes without hearing protection. All reported times when they had a temporary dulling of hearing and/or tinnitus, consistent with potentially damaging noise exposure (Brungart et al., 2019). All but two reported currently having tinnitus.

### Analysis Method

The steps in the analysis of the data were:

1. For each audiometric frequency, *f*, (0.5, 1, 2, 3, 4, 6, and 8 kHz) and each ear separately, the end-of-service HTL, HTL_EOS_(*f*), was subtracted from the HTL obtained at least 5 years later, denoted HTL_final_(*f*).
2. The difference obtained in step (1) was converted to the rate of change of HTL in dB/year by dividing the difference by the elapsed time in years between the the end-of-service audiogram and the later audiogram. This rate of change is denoted R_actual_ (*f*).
3. The expected audiogram for a man at the 50^th^ percentile based on ISO 7029 (2017) was calculated for the age at end of service and the age at the date of later audiogram and the difference between the two was calculated.
4. The difference obtained in step (3) was converted to the expected rate of change of HTL in dB/year by dividing the difference by the elapsed time in years between the the end-of-service audiogram and the later audiogram. This rate of change is denoted R_expected_(*f*).

If R_actual_ (*f*) is greater than R_expected_(*f*), this indicates an accelerated progression of hearing loss following the end of military service. If R_actual_ (*f*) is less than R_expected_(*f*), this indicates a slowing of the progression of hearing loss following the end of military service. If R_actual_ (*f*) is equal to R_expected_(*f*), this indicates that the noise exposure during military service had no effect on the subsequent progression of hearing loss.

### Results

**Figure 2.**
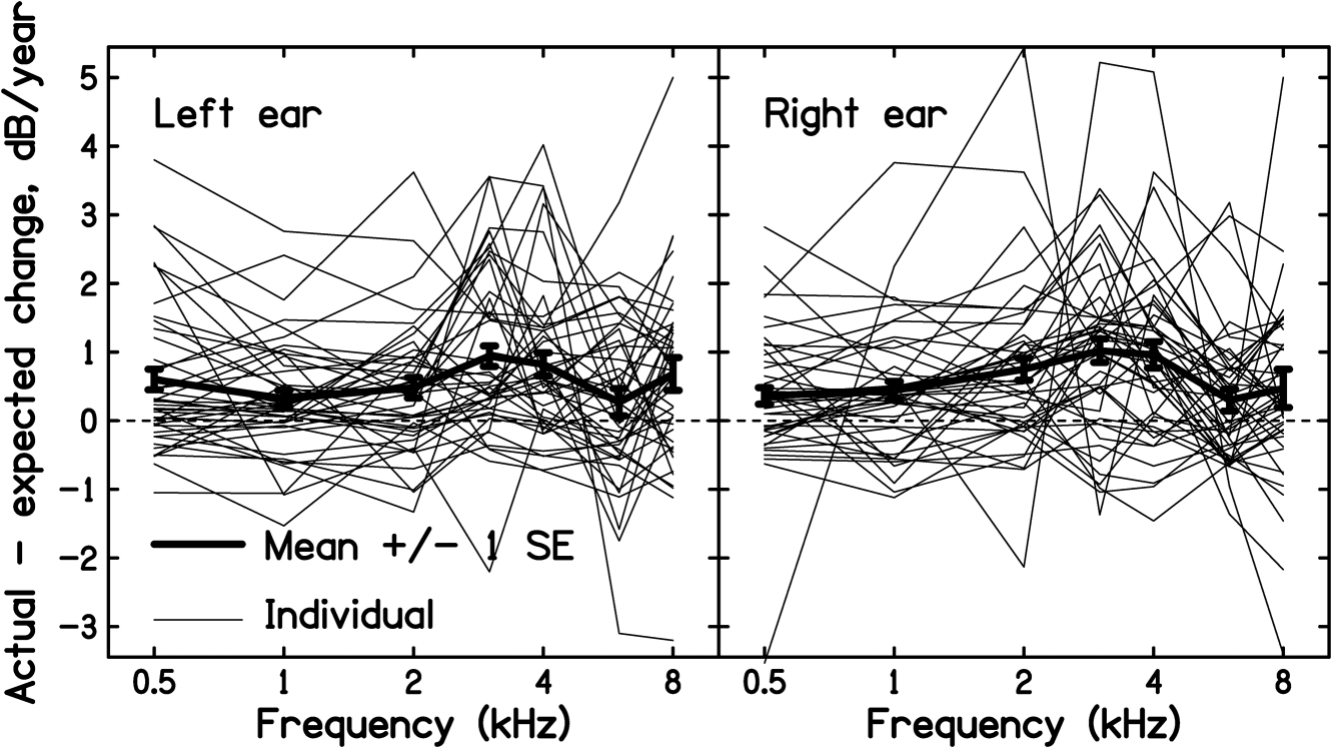
Difference between the actual rate of change of HTL and the rate of change expected from ISO 7029 (2017), plotted as a function of frequency. Thin lines show the individual results and thick lines show the mean. The left and right panels shows results for the left and right ears, respectively. The error bars show ± 1 standard error (SE) of the mean.

The individual and mean values of R_actual_ (*f*) − R_expected_(*f*) are shown separately for each ear in Figure 2. It is clear that there was considerable individual variability. However, the mean value of R_actual_ (*f*) − R_expected_(*f*) (bold line in each panel) was above 0 for every audiometric frequency for both ears, indicating, on average, accelerated progression of hearing loss following the end of military service. The mean values of R_expected_(*f*), R_actual_ (*f*), and the differences R_actual_ (*f*) − R_expected_(*f*) are shown for each ear in Table 1. The differences from 0 were more than 2 standard errors (SEs), indicating significant acceleration for all cases, except for the left ear at 6 kHz. The difference was generally greatest for frequencies from 3 to 8 kHz, which are the frequencies for which HTLs are usually most affected by noise exposure during military service (Lowe & Moore, 2021; Moore, 2020). The small and non-significant acceleration for the left ear at 6 kHz probably reflects the fact that the HTLs at the end of service were on average greater for that case than for any other combination of frequency and ear; see the left panel of Figure 1.

**Table 1.** Average Expected Rates of Change of HTL, R_expected_(*f*), Average Actual Rates of Change of HTL, R_actual_ (*f*), and Differences Between Them, Shown Separately for Each ear and Each Frequency. Asterisks Indicate Significant Differences Between Actual and Expected Rates at *p* < 0.05.

		Frequency, kHz						
Ear		0.5	1	2	3	4	6	8
Left	Expected rate, dB/year	0.17	0.21	0.34	0.45	0.55	0.71	0.83
Left	Actual rate, dB/year	0.71	0.54	0.83	1.47	1.53	0.98	1.67
Left	Difference, dB/year	0.55*	0.32*	0.49*	1.01*	0.98*	0.27	0.84*
Right	Expected rate, dB/year	0.17	0.21	0.34	0.45	0.55	0.71	0.83
Right	Actual rate, dB/year	0.63	0.58	1.00	1.64	1.75	1.14	1.62
Right	Difference, dB/year	0.46*	0.37*	0.66*	1.19*	1.19*	0.43*	0.79*

**Figure 3.**
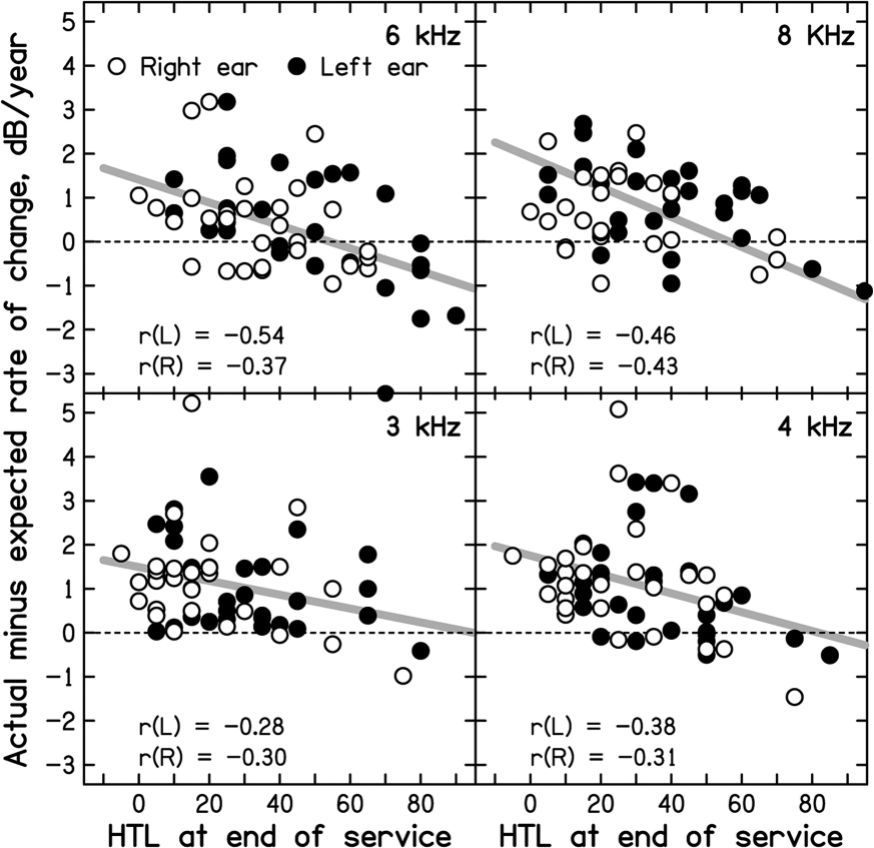
Difference between the actual rate of change of HTL and the rate of change expected from ISO 7029 (2017), plotted as a function of the HTL at the end of military service, HTL_EOS_(*f*). Each panel shows results for one frequency. Open and filled symbols show the data for the left and right ears, respectively. Correlations (r) are shown separately for each ear by the inset values in each panel. The gray lines are linear regression lines fitted to the data for the two ears combined for each frequency.

If the rate of progression of hearing loss decreases with increasing hearing loss at the end of military service, then R_actual_ (*f*) − R_expected_(*f*) should be negatively correlated with HTL_EOS_(*f*). Figure 3 is a scatter plot of values of R_actual_ (*f*) − R_expected_(*f*) against HTL_EOS_(*f*). Each panel shows results for one frequency. The correlations between R_actual_ (*f*) − R_expected_(*f*) and HTL_EOS_(*f*), shown in each panel, were small but were consistently negative, for all frequencies and both ears. Based on a directional hypothesis that the correlation should be negative, for a sample of 29 cases, a correlation should be more negative than −0.31 to be significant at *p* < 0.05. This condition was satisfied for both ears at 4, 6 and 8 kHz, but not at 3 kHz. Since the results were similar for the two ears (open and filled circles) for each frequency, a linear regression line was fitted to the data for both ears combined for each frequency (thick gray lines). The regression lines all had negative slopes, consistent with the hypothesis that the rate of decline in HTL decreases with increasing HTL_EOS_(*f*).

It is noteworthy that for values of HTL_EOS_(*f*) in the range −5 to 40 dB HL most values of R_actual_ (*f*) − R_expected_(*f*) were positive, i.e. for small values of HTL_EOS_(*f*) the actual rate of change was mostly greater than expected. In contrast, for values of HTL_EOS_(*f*) of 50 dB HL or more, the values of R_actual_ (*f*) − R_expected_(*f*) were roughly equally often positive and negative. This pattern of results is consistent with the hypothesis that for frequencies where HTL_EOS_(*f*) is small, exposure to noise during military service accelerates the subsequent progression of hearing loss, while for frequencies where HTL_EOS_(*f*) is above about 50 dB HL, the prior noise exposure has no effect on or slows the subsequent progression of hearing loss. The regression lines for *f* = 3, 4, 6 and 8 kHz had a mean value of 1.7 dB/year at HTL_EOS_(*f*) = 0 dB HL, indicating an acceleration of about 1.7 dB/year when there was no hearing loss at the end of military service.

A problem with the analysis described above is that the relationship between R_actual_ (*f*) − R_expected_(*f*) and HTL_EOS_(*f*) may partly occur because of random errors in HTL_EOS_(*f*). The value of HTL_EOS_(*f*) is used to calculate R_actual_ (*f*) (but with a − sign). Hence, if HTL_EOS_(*f*) is “too high” as a result of a random error, R_actual_ (*f*) will be “too low”, while if HTL_EOS_(*f*) is “too low”, R_actual_ (*f*) will be “too high”. This would tend to lead to negative correlations. There will also be random errors in the value of HTL_final_(*f*) but these will be unrelated to HTL_EOS_(*f*). To assess the magnitude of the correlations that would be expected from random errors of measurement in HTL_EOS_(*f*) and HTL_final_(*f*), the following analysis was performed.

The actual change in HTL from end of service to final is

(1) $$\mathrm{Chang}e_{\mathrm{actual}}(f)\;=\;\mathrm{HT}L_{\mathrm{final}}(f)-\mathrm{HT}L_{\mathrm{EOS}}(f).$$

The rate of change of threshold in dB/year is

(2) $$R_{\mathrm{actual}}(f)=\mathrm{Chang}e_{\mathrm{actual}}(f)/Y,$$

where Y is the number of years from end of service to final. The expected end-of-service HTL for a man at the 50^th^ percentile using ISO 7029 (2017) is denoted HTL(ISO)_EOS_(*f*) and the expected final HTL is denoted HTL(ISO)_final_(*f*). The expected change in HTL from end of service to final is

(3) $$\mathrm{Chang}e_{\mathrm{expected}}(f)=\mathrm{HTL}(\mathrm{ISO}{)}_{\mathrm{final}}(f)-\mathrm{HTL}(\mathrm{ISO}{)}_{\mathrm{EOS}}(f).$$

The expected rate of change of threshold in dB/year is

(4) $$R_{\mathrm{expected}}(f)=\mathrm{Chang}e_{\mathrm{expected}}(f)/Y.$$

To assess the negative relationship between R_actual_(*f*) − R_expected_(*f*) and HTL_EOS_(*f*) that would be expected based on random errors of measurement in HTL_EOS_(*f*) and HTL_final_(*f*), the effects of such errors were simulated assuming that R_actual_(*f*) is independent of HTL_EOS_(*f*) and that R_expected_(*f*) = R_actual_(*f*). The analysis was done separately for each ear and each frequency of the 29 cases. The steps were as follows:

1. The value of R_expected_(*f*) was set equal to the value of R_actual_(*f*).
2. For each frequency and each ear, the value of HTL_EOS_(*f*) was “jittered” to simulate a random error, by adding a random number drawn from a Gaussian distribution with a mean of 0 and a standard deviation of 3.8 dB. The value of 3.8 dB represents the standard deviation of the difference in HTLs for manual audiometry conducted by two different testers (Margolis et al., 2010). The result was then rounded to the nearest 5 dB, to reflect the practice when recording an audiogram. The resulting quantity is denoted HTL_EOS_(*f*, jittered).
3. A similar but independent jitter was applied to HTL_final_(*f*), giving HTL_final_(*f*, jittered).
4. The actual minus expected rate of change in HTL from EOS to final was calculated as
(5) $$\mathrm{Diff}(f)=[\mathrm{HT}L_{\mathrm{final}}(\;f,\;\mathrm{jittered})-\mathrm{HT}L_{\mathrm{EOS}}(\;f,\;\mathrm{jittered})]/Y-R_{\mathrm{expected}}(f).$$
5. For each frequency, the correlation was determined between Diff(f) and HTL_EOS_(*f*, jittered).
6. For each frequency, the slope of the best-fitting line relating Diff(f) to HTL_EOS_(*f*, jittered) was determined
7. Steps 5 and 6 were repeated for 40 realizations of the random applied jitters and the resulting correlations and slopes were averaged.

 Table 2 compares the mean correlations between Diff(*f*) and HTL_EOS_(*f*, jittered) with the measured correlations between R_actual_(*f*) − R_expected_(*f*) and HTL_EOS_(*f*). For frequencies from 3 to 8 kHz, and for both ears, the measured correlations were more negative than the correlations resulting from random errors of measurement in HTL_EOS_(*f*) and HTL_final_(*f*).

**Table 2.** Comparison of the Mean Correlations Between Diff(*f*) and HTL_EOS_(*f*, Jittered), Labelled “Simulated”, with the Measured Correlations Between R_actual_(*f*) − R_expected_(*f*) and HTL_EOS_(*f*), Labelled “Actual”.

	Frequency, kHz						
Ear	0.5	1	2	3	4	6	8
Left, simulated	−0.24	−0.25	−0.17	−0.08	−0.10	−0.08	−0.07
Left, actual	−0.52	−0.42	−0.35	−0.28	−0.38	−0.54	−0.46
Right, simulated	−0.28	−0.26	−0.10	−0.11	−0.12	−0.10	−0.16
Right, actual	−0.56	−0.50	−0.08	−0.30	−0.31	−0.37	−0.43

 Table 3 compares the mean slopes of the lines fitted to the values of Diff(*f*) as a function of HTL_EOS_(*f*, jittered) with the measured slopes of the lines fitted to R_actual_(*f*) − R_expected_(*f*) as a function of HTL_EOS_(*f*). For frequencies from 3 to 8 kHz, and for both ears, the measured slopes were markedly more negative than the slopes resulting from random errors of measurement. On average, the obtained slope was more negative than the simulated slope by a factor of 9.9 for the right ear and 14.8 for the left ear.

**Table 3.** Comparison of the Mean Slopes of the Lines Fitted to the Values of Diff(*f*) as a Function of HTL_EOS_(*f*, Jittered), Labelled “Simulated”, with the Measured Slopes of the Lines Fitted to R_actual_(*f*) − R_expected_(*f*) as a Function of HTL_EOS_(*f*), Labelled “Actual”.

	Frequency, kHz						
Ear	0.5	1	2	3	4	6	8
Left, simulated	−0.014	−0.014	−0.003	−0.004	−0.001	−0.001	−0.001
Left, actual	−0.073	−0.039	−0.023	−0.013	−0.022	−0.030	−0.033
Right, simulated	−0.016	−0.013	−0.006	−0.003	−0.004	−0.003	−0.001
Right, actual	−0.057	−0.057	−0.008	−0.018	−0.020	−0.021	−0.042

To assess how the simulated correlations and slopes would be affected by the magnitude of the assumed jitter, the simulations were repeated with the jitter increased to an unrealistically high value of 7.6 dB. For frequencies from 3 to 8 kHz, and for both ears, the measured correlations remained more negative than the correlations resulting from random errors of measurement and the measured slopes remained markedly more negative than the slopes resulting from random errors of measurement. It can be concluded that the negative measured slopes are not solely a consequence of random errors of measurement in HTL_EOS_(*f*) and HTL_final_(*f*).

**Figure 4.**
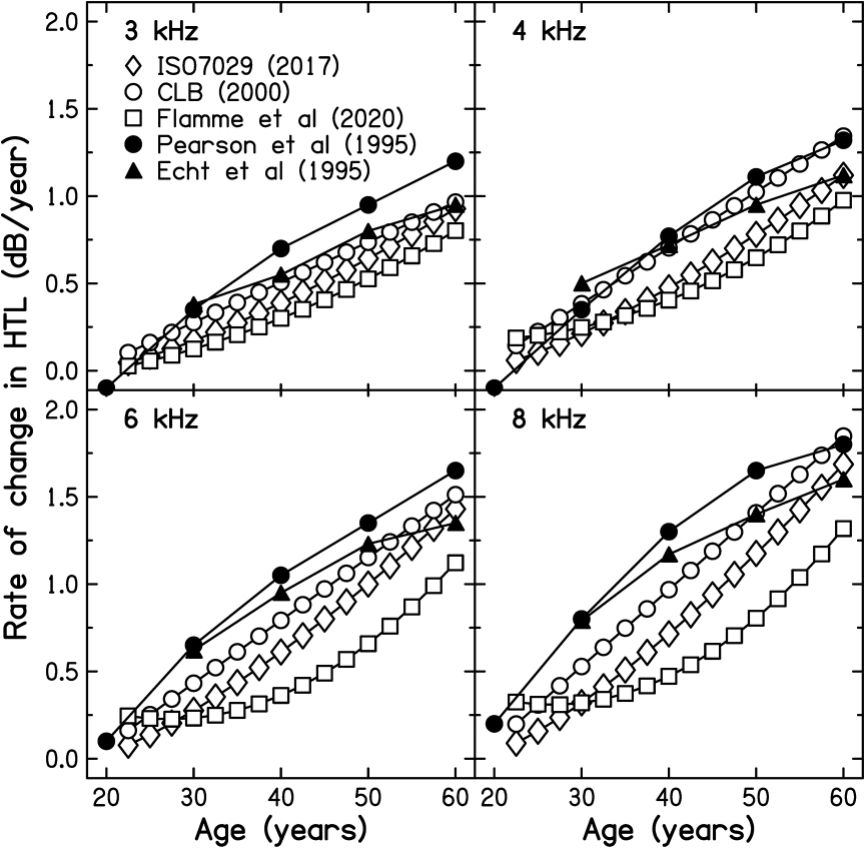
Rate of change of HTL as a function of age, estimated from three cross-sectional databases (open symbols) and two longitudinal databases (filled symbols), as indicated in the key. Each panel shows results for one frequency.

A possible objection to the analysis used to derive the results shown in Figures 2 and 3 is that the populations used to produce ISO 7029 (2017) were more carefully screened than the population of military veterans studied here. To assess the importance of the reference database used, rates of change of HTL were calculated for two databases with less rigorously screened populations, namely those of Coles et al. (2000), denoted CLB, and those of Flamme et al. (2020), using the 50^th^ percentile for the CLB data and the 75^th^ (best) percentile for the data of Flamme et al., as recommended by the authors (see below for more discussion of this point). The results are shown in Figure 4. For the majority of the population studied in the present paper, the relevant age range is from 25 to 45 years. For this range, the rates of change of HTL were greatest for the CLB database and smallest for the database of Flamme et al. (2020). However, for all three databases, the rates of change of HTL for those aged 35 years, the middle of the range that is relevant here, were equal to or less than 0.39, 0.54, 0.61 and 0.75 dB/year for frequencies of 3, 4, 6, and 8 kHz, respectively. These are all markedly less than the observed rates of change of HTL for small values of HTL_EOS_(*f*).

Another potential problem is that the three databases discussed above were all based on cross-sectional data; longitudinal trends were inferred from those data, although the results of Flamme et al. (2020) were verified using longitudinal data. Longitudinal trends inferred from cross-sectional data often differ from longitudinal trends measured directly (Humes, 2010). As noted by Flamme et al. (2020) “Cross-sectional trends are influenced by the combined effects of events (e.g. acute disorders, trauma, infection) and conditions that might be rare on the individual level (e.g. hereditary/genetic disorders) but have a collective impact on the distribution of hearing thresholds at the population level. These effects would be increasingly potent as a function of increased time at risk (i.e. correlated with age, but not an inexorable effect of age). The effects would be minimal on the tail of the distribution with better hearing sensitivity and would increase as consideration moves to the opposite tail of the distribution.” That was the reason why Flamme et al. (2020) recommended the use of the 75^th^ percentile to represent longitudinal trends.

There are only a few studies of longitudinal changes in HTLs for younger people with little noise exposure. One relevant study is that of Pearson et al. (1995). They estimated longitudinal patterns of change in HTLs for 681 men and 416 women, all from the USA, with no evidence of otological disease, unilateral hearing loss, or NIHL. NIHL was diagnosed when the HTL at 3, 4 or 6 kHz was at least 15 dB greater than the HTL at both 2 and 8 kHz. The ages of the men ranged from 20 to 90 years and they were followed for up to 23 years. The data were fitted using a mixed-effects regression model. The fitted rates of change of HTL in dB/year are shown by the filled circles in Figure 4. The rates of change are slightly greater than for the three databases based on cross-sectional data, but are still close to or below 1 dB/year for ages in the range 25–35 years, i.e. below the observed rates of change of HTL in our sample of military veterans for small values of HTL_EOS_(*f*). It should be noted that while Pearson et al. (1995) excluded participants whose audiograms showed evidence for NIHL, they did not exclude any participants based on a history of noise exposure during work or leisure activities. Also, although the men had occupations “generally believed to have relatively little noise exposure”, some of them would have performed military service during the years covered by the survey (the year of entry to the study was between 1965 and 1991, and the Vietnam war lasted from 1955 to 1975). The method of diagnosing NIHL used by Pearson et al. (1995) probably “missed” some cases of military noise-induced hearing loss (Lowe & Moore, 2021; Moore, 2020). Hence, the rates of change in HTL estimated by Pearson et al. (1995) are probably greater than for a fully non-exposed population.

A study with a similar design and the same exclusion criteria as Pearson et al. (1995) but using a different population was conducted by Echt et al. (2010). Most (88%) of the 995 men in the sample were enrolled between the ages of 30 and 59 years. The rates of change of HTL based on the longitudinal data are shown as the filled triangles in Figure 4. Again the rates of change are below 1 dB/year for those enrolled at age 30 years. Although most of the men in the study did not work in noisy occupations, it was noted that more than 90% of the sample had served in the military, and 44% saw combat, so again the rates of change in HTL estimated by Echt et al. (2010) are probably greater than for a fully non-exposed population.

Another relevant study is that of Karlsmose et al. (2000). They assessed changes in HTLs over a five-year period for a Danish rural population aged 31–50 years. They did not present the data separately for each audiometric frequency. However, the average rate of change of HTL for the 85 men in the sample aged 31–35 years, averaged across the frequencies 3 and 4 kHz, was 0.5 dB/year (the same for the two ears). The average HTL at baseline for these men at 3 and 4 kHz was 7.5 dB HL. The rate of change of 0.5 dB/year probably over-estimates the rate of change for a non-exposed population since about 25% of their sample reported exposure to noise for 2 days or more per week sufficient for them to have to raise their voice in order to be heard. Despite this, the rate of change was smaller than observed in the present study for small values of HTL_EOS_(*f*).

Finally, a longitudinal study of Kim et al. (2019) is relevant. They assessed changes in HTL over a nine year period for Koreans screened to exclude otological diseases but not screened to exclude those with high noise exposure. For those enrolled in the study in their 20s and 30s, the mean estimated rate of change of HTL for men at 8 kHz was about 0.53 dB/year and the mean HTL at baseline was about 17 dB HL. Again, the rate of change found by Kim et al. (2019) is smaller than observed in the present study for small values of HTL_EOS_(*f*).

It can be concluded that, regardless of the reference database that is used, the data support the hypothesis that for frequencies where the NIHL at the end of military service is mild, exposure to noise during military service accelerates the subsequent progression of hearing loss.

## Discussion

The results of the analyses support the hypothesis that for frequencies where the NIHL at the end of military service is mild or absent, exposure to noise during military service accelerates the subsequent progression of hearing loss. In contrast, for frequencies where the NIHL is moderate or severe at the end of military service, the prior noise exposure has no effect on or slows the subsequent progression of hearing loss. This is consistent with existing data, as reviewed in the introduction (Kim et al., 2017; Macrae, 1971; Macrae, 1991; Moore, 2021; Xiong et al., 2014). These findings have important implications for the assessment of claims for compensation for the effects of noise exposure during military service. Some military personnel have near-normal audiograms close to the end of military service but develop hearing loss after several years have elapsed. It is often argued that the hearing loss at the time of the claim cannot be attributed to the effects of noise exposure during military service, because the audiogram obtained at the end of military service was near-normal. The present results indicate that this argument is not valid.

Some limitations of the study should be noted. Firstly, the men studied were all claiming compensation for NIHL incurred during military service. This sample may not be fully representative of the general population of military veterans. Also, the sample studied was relatively small. It would be desirable to assess the hypothesis using a larger representative sample of military veterans.

Another limitation is that there was no control group of age-matched non-exposed men with similar demographics. Rather, the rate of change of HTL expected for each man in the sample was estimated using the 50^th^ percentile in ISO 7029 (2017). It might be argued that the populations on which ISO 7029 (2017) was based were more carefully screened than the sample studied in this paper. For example, the audiograms for people who reported the use of hearing protection were excluded from ISO 7029 (2017). However, for men in the age range 25 to 45 years, which applies to the majority of the sample studied in this paper, the observed rates of change of HTL with increasing age for those with near-normal HTLs at the end of military service were greater than expected based on several other databases (Coles et al., 2000; Echt et al., 2010; Flamme et al., 2020; Karlsmose et al., 2000; Pearson et al., 1995). Furthermore, as discussed earlier, two of these other databases included some men who had performed military service. It seems likely that for non-noise exposed men aged 30–40 years and with audiometric thresholds below 10 dB HL, the expected rate of change of HTL is 0.5 dB/year or less for frequencies from 3 to 8 kHz. This is markedly smaller than observed here for noise-exposed men with audiometric thresholds in the range −5 to 10 dB HL at the end of military service.

The reason for the accelerated progression of hearing loss following the end of noise exposure when the hearing loss at the end of military service is mild or absent is not clear. A possible explanation comes from the concept of a “cochlear reserve”, as described in the introduction. Studies of animals have shown that a certain amount of damage to the OHCs can occur with little or no change in the threshold for detecting sounds (Dallos & Harris, 1978; Evans & Harrison, 1976; Harrison & Evans, 1979). Consistent with this, measures of DPOAEs, which are thought to reflect the integrity of the OHCs, decline with increasing age from 30 years onwards and these declines are not matched by changes in audiometric thresholds (Glavin et al., 2021; Poling et al., 2014). Glavin et al. (2021) concluded that cochlear decline begins in the third decade of life, is greatest at the cochlear base, and cannot be detected fully by the audiogram.

A common consequence of noise exposure is tinnitus (Griest-Hines et al., 2021; Yankaskas, 2013). Tinnitus is thought to be associated with damage to the cochlea, even for those with normal audiograms (Schaette & McAlpine, 2011). Job et al. (2007) tested normal-hearing pilots aged 25–35 years with 8 ± 5 years of aircraft noise exposure, of whom 23% reported tinnitus after flight missions while 77% did not. The group with tinnitus had lower DPOAEs in the frequency range 1.5 to 2.8 kHz than the group without tinnitus. However, there was no difference between groups in their HTLs for frequencies up to 3 kHz. Job et al. (2007) concluded that their study provided evidence of OHC dysfunction in subjects with normal audiograms who had been exposed to noise and were susceptible to tinnitus.

The concept of the cochlear reserve probably applies also to the function of IHCs/synapses/neurons, but in a different way. Substantial damage to the IHCs, or to the synapses between the IHCs and the neurons that make up the auditory nerve, can occur with little effect on the detection threshold (Lobarinas et al., 2013; Sergeyenko et al., 2013). Probably, only a very few IHCs/synapses/neurons are sufficient to allow detection of a sound (Oxenham, 2016; Vinay & Moore, 2010). Hence, the audiogram is likely to be almost unaffected by loss of function of IHCs/synapses/neurons until the loss becomes very severe. Noise exposure can accelerate the progression of synaptopathy with increasing age (Fernandez et al., 2015), and after some time this may lead to sufficient loss of IHCs/synapses/neurons to affect audiometric thresholds.

Overall, these findings suggest that the cochlea has a certain “spare capacity” and can sustain some damage with only a small or no effect on the audiogram. However, once the reserve is sufficiently depleted, further minor damage associated with aging may produce a substantial worsening in the audiogram. It is also possible that depletion of the cochlear reserve is partly responsible for the frequent occurrence of problems in understanding speech in noise among former military personnel with normal or near-normal audiograms (Billings et al., 2018; Grant et al., 2021).

Individual variability in the progression of hearing loss following the end of military service was substantial. This partly reflects errors of measurement of the HTLs. However, some individuals with little or no hearing loss at the end of military service showed a marked acceleration of the subsequent progression of hearing loss for most audiometric frequencies, while others showed little or no progression for any frequency. The origin of these large individual differences is unknown.

The data and analyses presented in this paper were exclusively concerned with the effects of noise exposure during military service. Such noise exposure typically includes both intense impulsive sounds, such as rifle shots and the sound of artillery fire or mortars, and more steady sounds, such as vehicle and aircraft noise (Jokel et al., 2019). It remains unclear whether exposure to intense steady sounds, as occurs in some factories, can also lead to an acceleration of the progression of hearing loss after the exposure has ceased. Studies with mice support this possibility (Fernandez et al., 2015; Kujawa & Liberman, 2006). However, those studies used relatively short duration exposures (2 h), which were sufficiently intense to produce synaptopathy. It is not known whether longer-term exposure to moderately intense steady noise can lead to an accelerated progression of hearing loss. Also, while impulsive sounds appear to be more damaging to the ear than steady sounds with the same energy (Zhang et al., 2021), it is not known whether exposure to moderately intense impulsive sounds (e.g. hammering) is more likely to accelerate the progression of hearing loss than exposure to steady sounds with the same overall energy. Finally, it is not known whether exposure to intense sounds at rock concerts or discotheques has the potential to accelerate the progression of hearing loss with increasing age. More research on these issues is clearly needed.

## Summary and Conclusions

This paper tested the hypothesis that for frequencies where NIHL at the end of military service is mild or absent, exposure to noise during military service accelerates the subsequent progression of hearing loss. In contrast, for frequencies where the NIHL is moderate or severe at the end of military service, the prior noise exposure has little or no effect on or slows the subsequent progression of hearing loss. The analysis was based on new longitudinal data obtained from 29 former military personnel, all men. Audiograms obtained at the end of military service were compared with those obtained at least five years later. Rates of change of HTL in dB/year were compared with those expected from ISO 7029 (2017) for a man at the 50^th^ percentile and with those expected from several other databases. The results suggest that noise exposure during military service accelerates the progression of hearing loss for frequencies where the hearing loss is absent or mild at the end of military service, by about 1.7 dB/year on average for frequencies from 3 to 8 kHz, but has no effect on or slows the progression of hearing loss for frequencies where the HTL exceeds about 50 dB HL. Acceleration, when present, appears to occur over a wide frequency range, including 1 kHz, consistent with the data of Macrae (1971, 1991).
